# User Requirements for Technology to Assist Aging in Place: Qualitative Study of Older People and Their Informal Support Networks

**DOI:** 10.2196/10741

**Published:** 2018-06-06

**Authors:** Phoebe Elers, Inga Hunter, Dick Whiddett, Caroline Lockhart, Hans Guesgen, Amardeep Singh

**Affiliations:** ^1^ School of Management Massey Business School Massey University Palmerston North New Zealand; ^2^ School of Engineering and Advanced Technology College of Science Massey University Palmerston North New Zealand

**Keywords:** aging in place, informal support, smart home, home monitoring technology

## Abstract

**Background:**

Informal support is essential for enabling many older people to age in place. However, there is limited research examining the information needs of older adults’ informal support networks and how these could be met through home monitoring and information and communication technologies.

**Objective:**

The purpose of this study was to investigate how technologies that connect older adults to their informal and formal support networks could assist aging in place and enhance older adults’ health and well-being.

**Methods:**

Semistructured interviews were conducted with 10 older adults and a total of 31 members of their self-identified informal support networks. They were asked questions about their information needs and how technology could support the older adults to age in place. The interviews were transcribed and thematically analyzed.

**Results:**

The analysis identified three overarching themes: (1) the social enablers theme, which outlined how timing, informal support networks, and safety concerns assist the older adults’ uptake of technology, (2) the technology concerns theme, which outlined concerns about cost, usability, information security and privacy, and technology superseding face-to-face contact, and (3) the information desired theme, which outlined what information should be collected and transferred and who should make decisions about this.

**Conclusions:**

Older adults and their informal support networks may be receptive to technology that monitors older adults within the home if it enables aging in place for longer. However, cost, privacy, security, and usability barriers would need to be considered and the system should be individualizable to older adults’ changing needs. The user requirements identified from this study and described in this paper have informed the development of a technology that is currently being prototyped.

## Introduction

Since the 1990s, there has been a move to promote “aging in place”, in which individuals continue living in their place of residence in later life [1]. Aging in place is preferred by most older people [2,3] and is seen as a way to maintain autonomy and connection with friends and family who provide both practical and emotional support [4]. Moving into residential care can be detrimental for older people, as it can lead to increased immobility and a loss of independence [5]. At the same time, there is growing recognition that neighborhoods and communities are crucial for enabling older people to age in place [2,6].

Various technologies have been used to support older people as they age in place, including home monitoring devices [7], purpose built smart homes [8], intelligent cognitive assistants [9], and online health information resources [10]. However, many technologies have the limitation of treating only one condition in isolation, rather than the older person as a whole, who may be dealing with a range of health issues. Additionally, there are various barriers to the uptake of these technologies among older people, including the cost, privacy concerns, and the perception that it is not required [11-13]. As older people are more likely to experience physical and cognitive decline associated with aging, this can also limit their ability to use technology [11,14].

Technologies used to assist older adults aging in place tend to be focused on providing communication pathways with formal health care providers, which neglects the older adults’ informal support networks of friends, neighbors, and family members who provide ongoing practical and emotional support such as personal care, housework, company, and emotional assistance [15]. This informal support is often under-recognized, even though it is often essential for enabling older people to age in place. A recent report determined that informal caregivers across Europe assume 50-90% of the responsibility for the long-term support of elderly, dependent people and most of them have limited access to formal support services [15]. According to Fischert et al [16], “Tools for the elderly should consider the whole care network and take into account who will be using the tool, who has access to what information, and how these factors may change over time” (p. 630). The involvement of friends and family members is also beneficial because these individuals can influence whether an older person will adopt a technology [11]. Accordingly, Luijkx et al [17] emphasized the importance of including family members, including grandchildren, when implementing technologies into the lives of older adults.

This paper presents some of the findings from an exploratory project that investigates how technologies that connect older adults to their informal and formal support networks could assist aging in place and enhance older adults’ health and well-being. The term “informal support networks” in this project refers to individuals identified by older adults as helping them age in place, which may include friends, neighbors, and family members, while “formal support networks” refers to organizations that provide health care services to the older adults. We present the findings from in-depth interviews with 41 participants, made up of 10 older adults and 31 members of their self-identified informal support networks. These participants were asked questions concerning their information needs and how technology could support older adults to age in place. The findings from focus groups conducted with health care professionals working with older adults, which formed part of this study, will be presented in a separate paper.

The findings of this study identified a series of user requirements for the technologies that have informed the development of a technology currently being prototyped in a later phase of the project. The work reported here adds to the scholarly body of knowledge by examining how technologies that assist older adults in their residences could be tailored to end users and how reported barriers could be overcome. This study is significant because the information needs of older adults’ informal support networks have been largely neglected in health informatics research to date.

## Methods

### Overview

Semistructured interviews were conducted with older adults and members of their informal support networks between March and June 2017. In-depth interviews were selected because they can allow for an in-depth understanding of participants’ perspectives of a chosen phenomenon [18]. Research ethical approval was obtained from the Massey University Human Ethics Committee (SAO 16/65).

### Recruitment

Older adults were recruited using convenience sampling of individuals who indicated an interest in participating in the study. Community organizations were contacted about the study and asked to display posters on notice boards and distribute information to potential participants. Interested participants then contacted researchers directly. The older adults were required to be aged 70 years or older, live alone in the Manawatu region in New Zealand, and have at least one chronic health condition. They were excluded if they lived in a retirement village or in residential care. These criteria were selected so that the older adults recruited were likely to be nearing the stage of requiring assistance to age in place. Participating older adults additionally needed to identify at least 3 members of their informal support networks of friends, neighbors, and family members who provide them with ongoing practical and emotional support and were willing to participate in the study.

Ten older adults and 31 informal support network participants were recruited. The older adults were aged 74-92 years; 8 were female and 2 were male. The support network participants ranged in age from 22-80 years old; 25 were female and 6 were male. They included the older adults’ family members (siblings, children, and grandchildren), friends, and neighbors who were providing practical and/or emotional support.

### Interview Design and Content

The interviews with the older adults were face-to-face, while the interviews with the support network participants were a mixture of face-to-face, over the phone, and via Skype. They were all conducted by two members of the research team. The face-to-face interviews took place at public places where privacy could be maintained. A semistructured interview design was used [18], which meant that the interviews could focus on the subject at hand, while still allowing for some spontaneity and expansion on complex issues.

An interview guide with probes was developed to identify information needs and how these could be met through technologies, partly guided by a workshop conducted with participants attending a health informatics conference in 2016 [19]. The interviews focused on home monitoring and information and communication technologies, as the study’s goal is on enhancing older adults’ wellness and assisting aging in place, rather than treating medical conditions. The interview questions were piloted during the development stage and are provided in Multimedia Appendix 1.

### Analysis

The interviews were transcribed and then anonymized with unique identifiers that linked the older adults to their support network participants. The transcripts were thematically analyzed [20] manually in NVivo Version 11.0. The coding and theme development was inductive, using an iterative process that involved reading and rereading the datasets to establish initial codes that covered key ideas discussed and then combining similar codes under themes. Following this process, the themes were reviewed alongside the original dataset. During the analysis, the codes and themes were discussed within the research team and finalized.

## Results

The analysis of the interviews identified three overarching themes: (1) social enablers, (2) technology concerns, and (3) information desired. Each theme has several subthemes described below.

### Theme 1: Social Enablers

Almost all the participants expressed an acceptance of some home monitoring technology if it were needed to allow the older adults to age in place and avoid residential care. This theme includes their descriptions of factors that help increase the uptake of home monitoring and information and communication technologies among the older adults. As the title of this theme suggests, many of these concerns were related to the older adults’ social well-being, as opposed to specific medical conditions. For instance, some older adults discussed how they already use a range of digital technologies, such as email, text messaging, Skype calls, and social media sites to connect with members of their informal support network and how this network has helped them use technology. This theme encompasses participants’ discussions about how timing, the informal support network, and safety concerns can assist the uptake of technology among the older adults.

Timing and the acceptance of technology were closely related for both the older adult and support network participants. While some participants thought that technology would already be useful to assist aging in place, many others felt that it should be used in the future when required. A number of support network participants thought that technology could assist them in monitoring the older adults in the future. One participant stated:

If it came to the stage where I did have to look after her that would be when I would want to “technology-up” with the sensors and that.

daughter of Older Adult 9, 45-year-old female

Another stated:

I would consider that [technology] if we were trying to keep Mum at home because she had advanced cognitive issues.

daughter of Older Adult 3, 59-year-old female

The older adults’ informal support networks were another enabler for their uptake of technology. For some older adults, technology was a way of connecting with members of their support network in modern terms, sometimes at a geographical distance. Many support network participants actively encouraged the older adults to use technology and assisted them in doing so. One participant described her experience in helping the older adult use a mobile phone, stating:

It was quite a lot of perseverance getting her to use it. But…she’s got grandkids overseas and my aunt and uncle overseas and so she texts them and it’s great.

granddaughter of Older Adult 2, 29-year-old female

An older adult discussed how her son remotely assists her in using technology, stating:

When I wanted some help with the computer…he said, “invite me on to your computer” and I would sit there, and he would fix the computer from California while I looked on.

Older Adult 8, 77-year-old female

However, some older adults raised concern about disturbing or burdening their support network. As one participant stated:

My network people or my neighbors are all busy rushing around doing whatever they want to do. They might not be there

Older Adult 5, 83-year-old female

Many participants considered technology to be a way to monitor and maintain the older adults’ safety within the home, which was presented as being of high significance. In some cases, technology was already used for monitoring the older adult’s well-being, such as with the support network member texting or emailing the older adult daily for a welfare check. Some older adults even indicated that they would be willing to forego what they would consider an invasion of privacy to keep themselves safe at home and avoid residential care. For instance, the possibility of falling seemed to change one older adult’s mind about having cameras in the home as indicated in the following quotation:

I don’t think I’d like a camera following me around all day! But then, if you’re falling and you can’t, well, push your [emergency] alarm.

Older Adult 2, 85-year-old female

### Theme 2: Technology Concerns

Although most participants accepted the idea of technology being used to assist the older adults when required, there were concerns raised about some possible functions of the technology, how it would be managed, and the social consequences. The main issues raised were cost, usability issues, information security and privacy, and fears that technology could supersede face-to-face interpersonal communication.

A significant, and perhaps the strongest, concern expressed by older adults was the cost of the technology. For many older adults, finances already hindered their uptake of technology. This was acknowledged by many support network participants. As one stated, “Cost of anything is a problem to [Older Adult 10]” (friend of Older Adult 10, 46-year-old male). Similarly, a support network participant of Older Adult 6 stated:

Oh, actually, that [the cost] could be a huge factor. One of the main ones.

granddaughter of Older Adult 6, 21-year-old female

Another participant discussed the issue of the cost of technology for elderly more generally, stating:

A lot of elderly people, they’ve got very little money…who’s going to pay for it?

daughter of Older Adult 3, 56-year-old female

Usability issues were strongly raised by both the older adult and support network participants. Overall, the older adults were more concerned with whether technology would work within their home and lifestyle, while the support network participants were concerned with the older adults’ ability to use technology. A few support network participants commented that they had observed older adults worsen in conditions associated with aging, such as vision and dexterity impairment, which might reduce or limit the older adults’ ability to continue to use the technology. As one participant stated:

Older people losing their sight, losing their hearing, both of which worsen with age, is a real challenge for digital, because what’s okay to start with may not be okay a year later.

daughter of Older Adult 5, 60-year-old female

The most common ethical issue raised was the security and privacy of personal information. Some participants did not seem to trust the security of information systems and raised concerns that individuals could access information without gaining informed consent from the older adults. Significantly, this was raised more strongly by the support network participants. For instance, one participant stated:

You have to trust somebody, and if St John’s comes and takes you off to hospital, you have to trust that they’re not going to tell the neighborhood that that house is empty…you have to trust somebody, but somebody up in the cloud?

friend of Older Adult 3, 75-year-old female

Additionally, both the older adult and support network participants expressed concern that technology could inadvertently replace face-to-face interaction. For example, support network participants described how they check on the older adults’ well-being during visits, such as their mood and the temperature of the home, and so if technology could electronically replace this, these visits could become less pressing and decrease. This is significant as many participants, particularly those that were older, emphasized that technology does not facilitate communication of the same depth compared to face-to-face interaction. As one participant stated:

You can have one of those devices and that will tell [name] or [name] or some guy what’s happening, but they’re not close enough to make her a cup of tea.

friend of Older Adult 3, 75-year-old female

### Theme 3: Information Desired

A crucial topic discussed by the participants was how much information should be collected so that the technology is both useful and does not impinge on the older adults’ privacy. Views about this were diverse, although there was consistency among many participants in the idea of the information being processed and the technology being individualized to the older adult. This theme includes discussions about what information should be collected and transferred and who should make decisions about this information.

The information that the participants wanted to be collected and transferred varied significantly. Some support network participants wanted to receive a large quantity of information, such as a granddaughter (29-year-old female) who wanted to be notified each time that Older Adult 2 (85-year-old female) has a medical appointment, the outcome of each medical appointment, information about her diet, and her whereabouts. Others preferred to receive only information that they considered to be of a serious nature, such as a daughter (56-year-old female) who wanted to be notified when Older Adult 3 (81-year-old female) is in hospital, her condition after surgery, and information about serious falls. There were some older adults who were accepting of any information being transferred, while others, like the support network participants, wanted only information of a serious nature transferred. For instance, one older adult stated:

Well, if I’ve had a fall in the middle of the street somewhere and I’ve grazed my leg or something and I’m not trotting off to hospital, no, I wouldn’t want her notified about that. But if the ambulance has come and I’ve knocked my head and I’ve lost consciousness or something, that might be a good idea!

Older Adult 1, 82-year-old female

However, many participants thought that information should be transferred only when there is a change in the older adults’ usual routine. For example, rather than notifying the support network when the older adult gets out of bed in the morning, they would be notified only if the older adult is not up when it is past their usual time. This idea of processing the information before it is transferred was popular among all the participants—it was considered less intrusive for the older adults and the informal support networks would have less information to sort through. One support network participant stated:

I don’t need to know that she’s opened the fridge 10 times today, like if she hasn’t opened the fridge at all today then I do…and then also if the fridge door stays open the whole time.

daughter of Older Adult 5, 60-year-old female

With a few exceptions, the participants thought that the older adults should decide what information is collected and transferred and to whom. Although some support network participants wanted more information than the older adult would be willing to have collected, they still thought that the technology should be individualized to the older adults’ self-determined needs. On the whole, the older adults wanted to be in control of how the technology would be used and managed. As one older adult stated:

I would want control, yeah. Yeah, somebody told me once that I like being in control! And it’s true. Don’t we all?

Older Adult 3, 81-year-old female

## Discussion

### Principal Findings

The work reported in this paper is part of the first phase of an exploratory project that investigates how technologies that connect older adults to their informal and formal support networks could assist aging in place and enhance older adults’ health and well-being. Semistructured interviews were conducted with 10 older adults and 31 members of their self-identified informal support networks. The limited size of the study means that care should be taken not to overgeneralize the conclusions, but several common themes emerged within the findings. Overall, participants were accepting of the idea of technologies that monitor older adults in the home when required to enable aging in place.

However, concerns were raised that could hinder the uptake of this technology, pertaining to cost, usability, information security and privacy, and fears that it could supersede face-to-face interpersonal communication. This aligns with research that has demonstrated that older people have concerns about cost and privacy regarding these technologies [11-13] and that physical and cognitive decline associated with aging can limit their ability to use technology [11,14].

To date, health informatics scholarship has largely overlooked the role of informal support networks for aging in place and how their information needs could be assisted through technologies within the home. This study has shown that older adults’ informal support networks want more information about the older adults’ well-being, and in many cases older adults are accepting of certain home monitoring technologies transferring this information if it would allow aging in place longer. A number of support network participants already use available information and communication technologies to monitor the older adults on a daily basis, such as email or text messaging as a form of welfare check. Furthermore, support network participants actively encourage the uptake of technology among the older adults, aligning to existing literature that has determined that friends and family members influence whether an older adult will adopt a technology [11]. From the analysis, several user requirements can be deduced for a technology that assists older people to age in place. A list of high-level user requirements for the technology that have been derived from the analysis themes are summarized in Table 1 and discussed below.

First, as much as possible, the technology needs to be low cost. The concern about financial barriers was strongly expressed by most of the older adults interviewed. One way to achieve this could be to use technologies that already exist within the home, including televisions, computers, and home appliances that, with modification, transfer information about the older adults’ well-being. This could also help address some of the older adults’ concerns about the technology being adaptable to their home and lifestyle.

**Table 1:** User requirements derived from the analysis themes.

Theme and subthemes		Number	User requirement
**Social enablers**			
	Compatibility	1	The technology needs to be easy to install.
	Compatibility	2	The technology needs to be adaptable to different home layouts.
	Compatibility	3	The technology should be adaptable to meet older adults’ changing needs.
	Usefulness	4	The technology should save users’ time.
	Compatibility	5	The technology should be able to use a variety of technologies to connect older adults to members of their nominated support network.
**Technology concerns**			
	Cost	6	The technology needs to be at a low cost.
	Cost	7	When possible, the technology should utilize objects already present within the home.
	Privacy	8	The information needs to be stored securely.
	Privacy	9	The security of the information needs to be clearly communicated to potential users.
	Usability	10	The interface needs to be stable and easy to navigate, with large text.
**Information desired**			
	Control	11	The information collected needs to be decided by the older adult.
	Control	12	Access to information should be limited to individuals nominated by the older adult.
	Control	13	It should be possible to send different information to different support network members.
	Processing	14	The technology should be able to send raw and processed data.
	Processing	15	It should be possible to only send information “outside of the norm” to the support network.

Second, the retrieved information needs to be stored and transferred securely. As the security of personal information was a concern raised by most of the participants, this should be clearly communicated to potential users.

Third, the technology needs to be designed for ease of use. It should be automated where possible. Any interface designed for the older adults should be stable and easy to navigate, with large text. For older adults with impairments that limit their ability to use technology, a direct user interface in their homes may not be required, the technology could simply monitor the environment so that the older adult would not need to actively engage directly with the system.

Fourth, the technology should be individualized to the older adults’ self-determined needs. Most of the participants emphasized that older adults should be in complete control of the technology. Thus, the older adults should determine what information is collected, sent, and who would receive this information. The technology should also be adaptable to meet the older adults’ changing needs, both in terms of the usability and the information collected and transferred. 

Finally, the technology should be able to process information so that nominated members of the support network may be notified only when the older adults act outside of their usual routine, such as when there is no movement in the home during certain hours or if the oven is on for an extended period. This may require initial monitoring to establish the older adults’ routine or it could be self-identified. As the participants discussed, the advantages of processing information in this way are twofold—it can be less intrusive for the older adults and less burdensome for the support network.

### Limitations

With the nature of qualitative research, the findings are limited to the 41 participants recruited and care should be taken not to overgeneralize the conclusions drawn from the study. Future research examining this topic should certainly take a wider scope. Nevertheless, the user requirements from this study have informed the development of a technology that is currently being prototyped. The intention is to assist aging in place and to enhance older adults’ health and well-being.

### Conclusions

While various home monitoring and information and communication technologies can support people in their homes [7,8,10], these are often underused [14]. With any technology, the user needs are paramount, and for older people these needs can be complex. The whole care network should be considered [14], which includes the informal support network, such as friends, neighbors, and family members. This study has identified that home monitoring and information and communication technologies could help connect older adults to their informal support networks to assist aging in place if specific barriers are overcome.

## Appendix

Multimedia Appendix 1 Interview questions.
