# Supplementary material for: User Requirements for Technology to Assist Aging in Place: Qualitative Study of Older People and Their Informal Support Networks
Source: JMIR Mhealth Uhealth. 2018 Jun 6;6(6):e10741. doi: 10.2196/10741 (PMC6010833; doi:10.2196/10741)
Supplement: Multimedia Appendix 1 [file mhealth_v6i6e10741_app1.pdf]

## Appendix: Interview Questions

### The older adults were asked:

1. How do you currently communicate with your support network?
2. What type of information about your health and wellbeing would you be comfortable to share and have sent to your support network to help you to stay living in your own home?
3. What information on your health and wellbeing would you not want to divulge in order to remain living in your own home?
4. What information would you find useful to receive?
5. How do you think information on your health and wellbeing could be collected?
6. Who would you want to receive information about your health and wellbeing?
7. Should there be a priority list of who gets notified if your condition changes?
8. How do you think the information of your health and wellbeing should be received?
9. Should the information on your health and wellbeing be processed after it is collected? And how?
10. Who should be responsible for the information that is collected and exchanged?
11. What ethical issues do you think could be raised by collecting, transfer and processing information on another person's health and wellbeing?
12. Is there anything else that you would like to add or discuss regarding this project?

### The support network participants were asked:

1. How do you communicate with [insert name here] and how often?
2. What type of information on [insert name here] health and wellbeing would you be comfortable receiving?
3. How would you like to receive information on [insert name here]?
4. What information would you find useful to receive?
5. How do you think the information on [insert name here] health and wellbeing could be sent and received?
6. Should the information on [insert name here] health and wellbeing be processed after it is collected? And how?
7. Who else should receive information about [insert name here] health and wellbeing?
8. Who should be responsible for the information that is collected and received?
9. What ethical issues do you think could be raised by collecting, transfer and processing information on another person's health and wellness?
10. Is there anything else that you would like to add or discuss regarding this project?
